# Android Pretending to Have Similar Traits of Imagination as Humans Evokes Stronger Perceived Capacity to Feel

**DOI:** 10.3389/frobt.2019.00088

**Published:** 2019-09-18

**Authors:** Kyohei Tatsukawa, Hideyuki Takahashi, Yuichiro Yoshikawa, Hiroshi Ishiguro

**Affiliations:** ^1^Department of Systems Innovation, Graduate School of Engineering Science, Osaka University, Osaka, Japan; ^2^JST ERATO Ishiguro Symbiotic Human Robot Interaction Project, Osaka University, Osaka, Japan

**Keywords:** robot, shared reality, mind perception, interpersonal closeness, visual imagination

## Abstract

The perception of robots as mindful enriches how humans relate to them. Given that congruence in perceived representations of the world enable humans to experience commonality in mental states (a shared reality), we propose that congruence between humans, and robots will encourage humans to attribute humanlike mental capacities to robots. To investigate this, we assessed the mental perceptions of a robot in a visual imagination task using Gray et al. mind perception scale, which evaluates experience (capacity to feel), and agency (capacity to plan and do). For each ambiguous picture in the designed task, humans, and a robot imagined an animal. The task was performed under six conditions (2 × 3: Lead/Follow for Low/Medium/High). In the Lead condition, the robot records its perceived animal first; in the Follow condition, the robot records after the human participant. The experiment had three different degrees of congruence: Low (0%), Medium (60%), and High (100%). The results showed that perceived experiences were higher in the Lead condition, suggesting that the robot is perceived to be empathetic. It is probable that the Follow condition was perceived as mimicry rather than shared reality. Therefore, the order of response may have played an important role in commonality in mental states. No differences were observed in the perceived agency across all conditions. These results suggest that the order of response affects how humans perceive the minds of robots. Additionally, we assessed a post-task questionnaire to evaluate the interpersonal closeness that the humans felt toward the android. The main effect was observed in the degrees of congruence. This result is in line with those of previous studies that report relationships across sharing of similarities and friendliness.

## Introduction

Human-like robots are increasingly becoming a part of society. These robots are utilized in various fields ranging from scientific studies (Mavridis, 2015) to performing actual human jobs (^1^ HUIS TEN BOSCH Co., Ltd., Sasebo, Japan). Application of robots with anthropomorphic appearance has captured the interest of many researchers. Some of these researchers focused on engineering applications, whereas others focused on testing psychological hypotheses (Złotowski et al., 2015).

A rapidly growing field of psychological study is how humans attribute anthropomorphic minds to robots to comprehend their intentions. There are many studies on how humans perceive the minds of robots. Gray et al. (2007) established the mind perception scale, which enables the perceived mind to be evaluated for two dimensions, namely, agency (capacity to plan and do), and experience (capacity to feel various emotions). Gray and Wegner (2012) demonstrated that the feeling of uncanniness toward a robot is related to the perceived experience. Stafford et al. (2014) investigated how the attitudes of elderly people toward robots and attribution of mind perception are connected. Studies such as the one by Salem et al. (2015) have also shown that robots that occasionally exhibit incongruent behavior are more likely to be perceived as mindful. Given that it is likely that human-like robots will disseminate further into modern society, it is important to continue considering the perceived minds of robots in a manner that is beneficial for enrichment of human–robot interactions (HRIs) and to determine ways to enhance the positive effects of such experiences.

In the present study, we consider the influence of shared reality on the perceived minds of robots. Simply stated, shared reality is the outcome of the process where the mental state of an individual perceives the world in a uniform manner (Echterhoff et al., 2009). Humans are known to have a strong desire to share their mental states with others (Hardin and Higgins, 1996; Higgins, 2005). Echterhoff et al. explained two motives for shared reality, namely, epistemic, and relational motives. Epistemic motive is the need to establish understanding of the world (Fu et al., 2007). Humans will refer to each other's realities to confirm the validities of their own realities (Byrne and Clore, 1967; Gross et al., 1995). Relational motive is the desire to forge interpersonal connections so as to positively impact emotional well-being (Diener and Seligman, 2002; Clark and Kashima, 2007). In either case, humans experience shared reality when there is a commonality among these shared mental states. Such commonalities in mental states are confirmed by checking mutual representations of certain targets of reference. We investigate whether experiencing such matched representations make humans feel shared reality even in HRI contexts. In other words, do humans feel that a robot has the same mental state when the representation it expresses is congruent with their own? When humans feel that the robot has the same mental state, the perception of mind toward the robot may alter in a manner similar to those in human traits. We hypothesize that humans will perceive a robot as more mindful when the robot expresses a similar representation.

We build upon Shinohara et al. and Tatsukawa et al. experimental paradigms to investigate how perceiving the same representations alter the perceived mind. Shinohara et al. paradigm was able to precisely control the degree of congruence by simply allowing the robot to repeat the human participants' responses to a question regarding their preferences (Shinohara et al., 2016). While this paradigm may simulate shared reality to some extent, it is possible that the human participants may interoperate the robot's response as a mimicry rather than the sharing of mental states. Mimicry is the act of merely matching one's behavior to that of another, and it does not always involve the sharing of mental states (Echterhoff et al., 2009). For example, Asch and Guetzkow have shown that a person will at times match his/her answer for a simple quiz to other members of the group (Asch and Guetzkow, 1951). The motive for matching the answer in this case is group pressure, rather than genuinely sharing a same representation. Tatsukawa et al. conducted a color perception task (Tatsukawa et al., 2018) to investigate shared perceptions. Human participants and a robot were required to answer the dominant color of a bi-colored image. Contrary to Shinohara et al.'s paradigm, they had the robot express its answer first, and the human participant followed thereafter. The drawback of this method was that the degree of congruence could not be precisely controlled. Thus, to eliminate the possibility of mimicry while maintaining full control over the degree of congruence, a different strategy is required.

In the present study, we devise an experimental paradigm to overcome these difficulties by hot reading, thereby enabling precise control over the degree of congruence in reality. Hot reading is a technique in which one secretly gains information about a certain person and subsequently uses this information during interactions. We therefore investigate how humans perceive a robot that seemingly shares a similar representation, using a visual imagination task. The humans and the robot are required to observe some ambiguous images (inkblot images used in the Rorschach test) together and speak the name of the animal that they individually perceived for each image. To eliminate the possibility of mimicry, the robot speaks its answer first in one of the conditions (Lead condition). We also assess the condition similar to Shinohara et al. work to observe whether mere repetition of the human participants' responses have any influence on the perceived mind (Follow condition). Using hot reading, the robot will be able to match or mismatch its imagined animal to that of the human participant at will, thus enabling precise control over the degree of congruence. Participants must then answer questionnaires regarding the perceived mind, and the answers are assessed to examine how the order of responses (Lead and Follow conditions), and the degrees of congruence (0, 60, and 100%) in representation affect the participants' perceptions toward the robot. Additionally, as sharing of reality is known to help establish social bonding (Hardin and Higgins, 1996; Hardin and Conley, 2001), we assessed its effect on feelings of interpersonal closeness toward the robot.

## Materials and Methods

### Participants

Participants were recruited by manually circulating flyers at the Toyonaka Campus of Osaka University and by posting a registration form on Twitter. Eighty-seven Japanese volunteers participated in the experiment (53 men and 34 women, mean age: 20.5 years, and SD: 2.1 years). Fourteen participants experienced the Low–Follow (9 men and 5 women, mean age: 20.6 years, and SD: 1.6 years), 14 experienced Medium–Follow (9 men and 5 women, mean age: 20.5 years, and SD: 1.0 year), 15 experienced High–Follow (9 men and 6 women, mean age: 20.5 years, and SD: 1.7 years), 14 experienced Low–Lead (8 men and 6 women, mean age: 20.5 years, and SD: 3.0 years), 15 experienced Medium–Lead (9 men and 6 women, mean age: 20.3 years, and SD: 1.9 years), and 15 experienced High–Lead conditions (9 men and 6 women, mean age: 20.8 years, and SD: 3.1 years). Three participants who noticed the presence of the secret apparatus were excluded from the analysis. (Detail is mentioned in the Result section.) All participants provided informed written consent prior to the experiment, which was approved by the Graduate School of Engineering Science of Osaka University.

### Measures

We assessed two psychological measurements, inclusion of the other in the self scale (IOS), and the mind perception scale. IOS is a single-item measurement for evaluating interpersonal closeness between a human and another participant (in this case, our android robot) (Aron et al., 1992). The interpersonal closeness is expressed by the degree of overlap between two circles that represent the self and the other. The human participant is requested to choose one of seven figures presented with different degrees of overlap.

The mind perception scale consists of 18 items that are categorized into two subscales: experience and agency (Gray and Wegner, 2012). In the current study, we created a questionnaire to evaluate these 18 items (e.g., Experience: “Do you think the android can feel pleasure?,” Agency: “Do you think the android can recognize emotion?”). Each item was evaluated on the basis of a five-point Likert scale. For example, if the participant was to rate the android's capacity to feel pain (item from experience category), they can answer from “not at all” (mark 1 on the answer sheet) to “extremely” (mark 5). The mean scores of the two subscales were calculated for each participant, and these scores were used to calculate the mean score of each group. Cronbach's alpha values for this study were α = 0.89 for experience and α = 0.84 for agency.

### Stimuli

Ten ambiguous images from the Rorschach test were prepared for this experiment. The images were displayed on a large screen using a software to control the precise duration of appearance of each image.

### Robot

**Figure 1:**
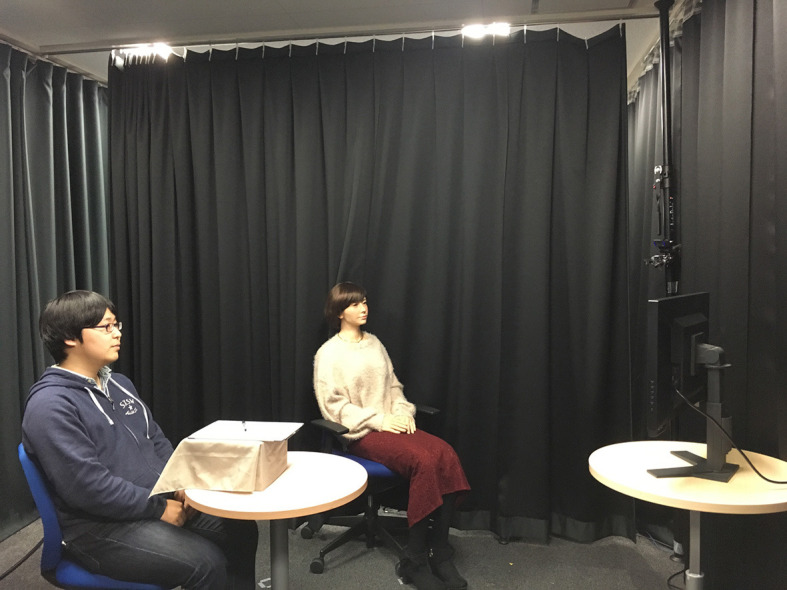
Experiment setting. The person to the left is the human participant. In the center is the android robot. To the right is the screen displaying the images. Written informed consent was obtained from the depicted individuals for the publication of these images.

We used Android-U, a female robot with an extremely human-like appearance for the experiment (Figure 1). Throughout the experiment, the robot would make slight neck motions and eye blinks at random times. Mouth motion while speaking was generated by a conventional method, which estimates the proper mouth motion based on the speech (Ishi et al., 2013). The phrases spoken by the robot were generated using text-to-speech software. The experimenter (the corresponding author of this work) determined the name of the animal necessary to fit the condition.

### Degree of Congruence in Representation

**Figure 2:**
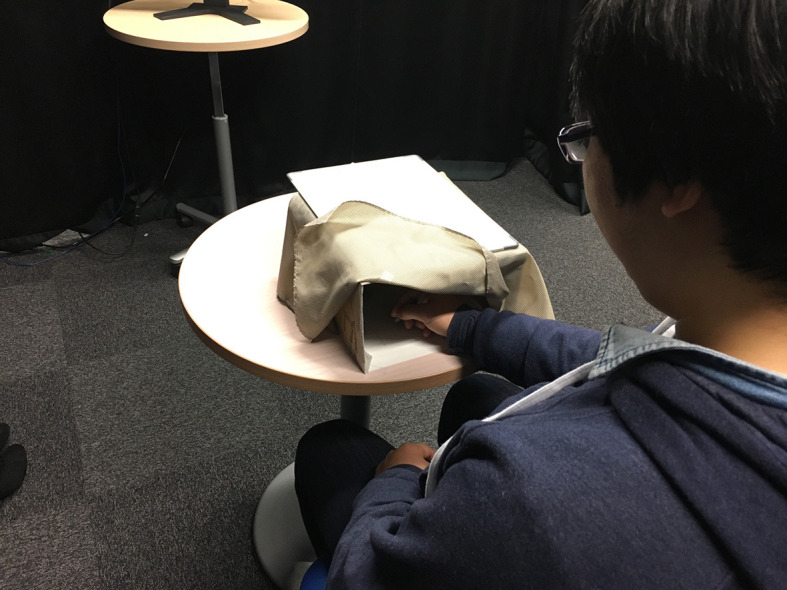
Participant writing down the name of the imagined animal. Written informed consent was obtained from the depicted individuals for the publication of these images.

To precisely control the degree of congruence in representation (in this case, the imagined animal), we utilized a spy camera to observe what animal the participant imagined. The spy camera is capable of sending live images through a wi-fi connection to a smartphone. Upon imagining the animal, the participants were instructed to write down the name of the animal they imagined on a sheet of paper hidden inside the box, as a record (Figure 2). The experimenter will then send the name of the participant's imagined animal to the text-to-speech software, thus enabling the android to match its representation with them. The experimenter can also send a different name so as to purposely mismatch the representation. For the Low–Follow and Low–Lead conditions, the experimenter sends mismatched names to all 10 images. For the Medium–Follow and Medium–Lead conditions, 6 matched and 4 mismatched names are sent. For the High–Follow and High–Lead conditions, 10 matched names were sent. The box acts as a shield to convince the participants that the robot cannot see what they wrote. The camera is attached to the upper portion of the box.

### Experimental Procedure

Following informed consent, the participants completed the visual imagination task, which consisted of a single session per participant. The experiment was conducted with one participant at a time.

**Figure 3:**
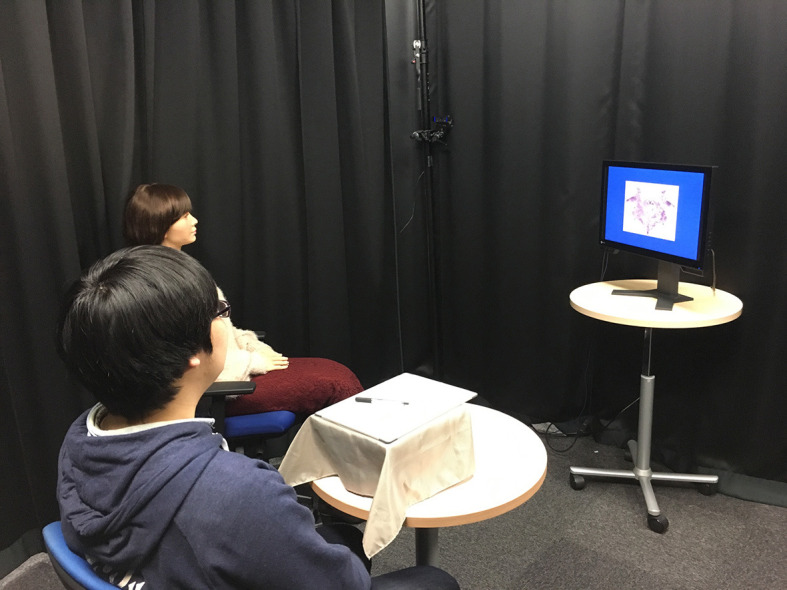
Participant and robot observing the ambiguous image. Written informed consent was obtained from the depicted individuals for the publication of these images.

For the Follow condition, a single participant was invited into the experiment room. The robot was introduced as having AI with a dedicated visual ability that closely resembles how a human perceives an object. The participant was then instructed to view 10 ambiguous images and imagine an animal for each case based on the image he/she saw. During the task, a fixation cross was displayed. The participant was told to concentrate on the screen and to closely examine the image because it would only be displayed for 10 s. After the image was removed from the screen, the program requested the participant to write down the animal that they imagined (Figure 3). Next, the program asked, “What did you see?” Requesting the participant to verbally answer first. The program then asked the robot to answer by prompting “How about you?” After the participants had responded, the program asked the participants to direct their attention to the screen for the next trial by announcing “The next picture will now be displayed. Please look at the screen.” This sequence is repeated, beginning with the fixation cross, until all 10 trials are complete. In the Lead condition, the procedure is exactly the same except the android answers first, followed by the participant.

To help the participants understand the task, the experimenter explained these abovementioned options to the participants in a step-by-step manner, prior to beginning the experimental trials. Once all participants understood the procedures, the experimental session began. After completion of the 10 trials, the participants were provided questionnaires (IOS and mind perception scale) for assessment. When the assessments were completed, the participants in the Follow conditions were asked whether they thought the android was merely mimicking their response. For the Lead conditions, the participants were asked if they suspected or noticed the presence of a hidden camera. Participants were then debriefed that the android was manipulated by the experimenter.

## Results

All but one participant from the High–Follow condition reported that they thought the robot was merely mimicking their response. Five participants from the Medium–Follow condition also reported similar sentiments. Three participants in the High–Lead condition suspected the presence of a hidden camera during the experiments. We conducted the analysis of the results by excluding these three participants (For raw data, please refer Data Sheet 1 in the Supplementary Material).

**Figure 4:**
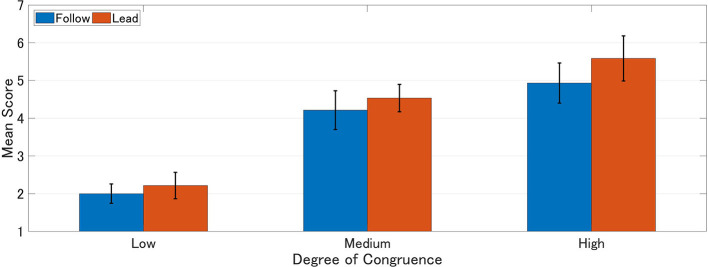
Mean IOS scores. Higher score represents stronger interpersonal closeness.

**Figure 5:**
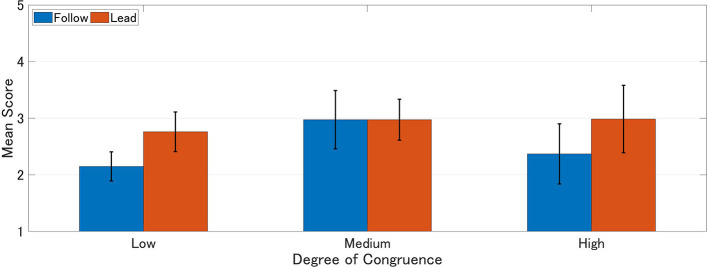
Mean score of perceived experience. Higher score represents higher perceived experience.

**Figure 6:**
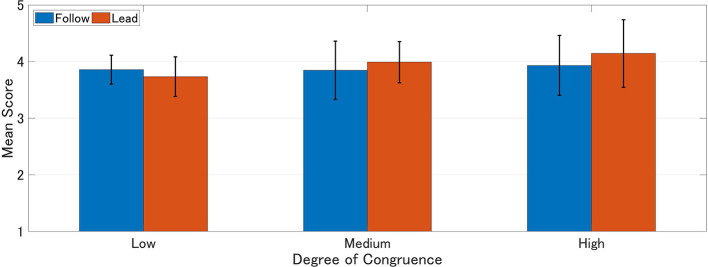
Mean score of perceived agency. Higher score represents higher perceived agency.

Mean scores of IOS, experience, and agency were calculated for each group. Higher scores represent higher interpersonal closeness, perceived experience, and agency (Tables 1–3). A 2 × 3 (order of response: Follow vs. Lead × degree of congruence: Low vs. Medium vs. High) ANOVA was performed on each of these scales (Figures 4–6).

**Table 1:** Descriptive statistics of IOS.

**Order of response**	**Degree of congruence**	**Mean**	**Std. deviation**	***N***
Follow	Low	2	0.926	14
	Medium	4.214	1.859	14
	High	4.933	1.982	15
Lead	Low	2.214	1.264	14
	Medium	4.533	1.36	15
	High	5.583	1.977	12

**Table 2:** Descriptive statistics of experience.

**Order of response**	**Degree of congruence**	**Mean**	**Std. deviation**	***N***
Follow	Low	2.15	0.706	14
	Medium	2.974	0.86	14
	High	2.37	0.686	15
Lead	Low	2.761	0.703	14
	Medium	2.823	0.683	15
	High	2.985	0.602	12

**Table 3:** Descriptive statistics of agency.

**Order of response**	**Degree of congruence**	**Mean**	**Std. deviation**	***N***
Follow	Low	3.858	0.645	14
	Medium	3.847	0.81	14
	High	3.933	0.784	15
Lead	Low	3.734	0.743	14
	Medium	3.99	0.677	15
	High	4.143	0.47	12

The ANOVA analysis of the IOS revealed main effect in the degree of congruence (*F*_(2, 81)_ = 26.27, *p* < 0.001, eta-squared = 0.400). It was found that medium congruence (Medium: *M* = 4.374, SD = 0.309) and high congruence (High: *M* = 5.258, SD = 0.323) were significantly higher in ratings than low congruence (Low: *M* = 2.107, SD = 0.315) (Medium vs. Low: *p* < 0.001, Cohen's *d* = 5.914) (High vs. Low: *p* < 0.001, Cohen's *d* = 8.109). On the other hand, order of response did not show a main effect, nor did it interact with congruence (*F*_(2, 78)_ = 0.13, *p* = 0.881) (Figure 4; Table 4).

**Table 4:** ANOVA of IOS.

	**Sum sq**.	***df***	**Mean sq**.	***F***	***p***	***η^2^***	**1-β**
A: Order of Response	3.249	1	3.2493	1.17	0.2824	0.0089	0.1376243
B: Degree of Congruence	145.705	2	72.8526	26.27	0	0.4002	0.9999997
A*B	0.703	2	0.3516	0.13	0.8811	0.0019	0.0617423
Error	216.298	78	2.773				
Total	364.036	83					

*df: degree of freedom, η^2^: effect size, 1-β: power*.

For experience, the ANOVA revealed that the main effect was observed in the order of response (*F*_(1, 82)_ = 4.90, *p* < 0.029, eta-squared = 0.05). Here, lead had significantly higher ratings (Lead: *M* = 2.856, SD = 0.113) than follow (Follow: *M* = 2.498, SD = 0.116) (Lead vs. Follow: *p* < 0.029, Cohen's *d* = 2.568). Contrary, no significant effect was seen for degree of congruence (*F*_(2, 81)_ = 2.56, *p* = 0.083), nor did it interact with degree of response (*F*_(2, 78)_ = 2.52, *p* = 0.086) (Figure 5; Table 5).

**Table 5:** ANOVA of experience.

	**Sum sq**.	***df***	**Mean sq**.	***F***	***p***	***η^2^***	**1-β**
A: Order of Response	2.6804	1	2.68037	4.9	0.0298	0.0528	0.57036
B: Degree of Congruence	2.8	2	1.39998	2.56	0.0838	0.0552	0.4798915
A*B	2.7612	2	1.38058	2.52	0.0867	0.0544	0.4735969
Error	42.6625	78	0.54695				
Total	50.7526	83					

*df: degree of freedom, η^2^: effect size, 1-β: power*.

The ANOVA did not show any significant main effect for agency scores (degree of congruence: *F*_(2, 81)_ = 0.75, *p* = 0.473) (response order: *F*_(1, 82)_ = 0.23, *p* = 0.633) (interaction: *F*_(2, 78)_ = 0.41, *p* = 0.666) (Figure 6; Table 6).

**Table 6:** ANOVA of agency.

	**Sum sq**.	***df***	**Mean sq**.	***F***	***p***	***η^2^***	**1-β**
A: Order of Response	0.1219	1	0.12194	0.23	0.6334	0.0029	0.0777235
B: Degree of Congruence	0.8022	2	0.4011	0.75	0.4737	0.0188	0.1829077
A*B	0.4341	2	0.21703	0.41	0.6663	0.0102	0.1179915
Error	41.4764	78	0.53175				
Total	42.7618	83					

*df: degree of freedom, η^2^: effect size, 1-β: power*.

## Discussion

### Summary and Implication

The purpose of this study was to investigate how humans perceived a robot that expressed the same representation. In the current experiment, an android robot and the participants were asked to observe some ambiguous images and verbally report their interpretations. The experiment was conducted under 6 conditions (degree of congruence: 0, 60, 100; order of response: robot report first or second). It was found that a higher degree of congruence contributed to a higher sense of interpersonal closeness. Perceived experience toward the robot was higher for the participants who mentioned their representation first, compared with those who expressed after the robot. Significant result was not seen for perceived agency.

From the results of IOS, our android was perceived to be more intimate when it matched the representation of the image with the participants' interpretation. Our results agree with those of a previous study, which states that a robot that expresses a similar attitude is perceived to be friendlier (Ono et al., 2001).

Differences in effects across the Follow and Lead conditions were observed for the perceived experience toward the robot, but not for the perceived agency. Participants who participated in the Lead conditions felt more experience compared with those in the Follow conditions, suggesting that exhibiting the robot's state prior to those of the human participants contributes toward enhancing the robot's capacity to feel. We speculate that the participants perceived weaker human-like mind toward the android in the Follow condition because the robot's response was predictable owing to its mimicry-like behavior. Previous research has suggested that incorporating unpredictability to a robot's behavior can facilitate its anthropomorphic aspect (Duffy, 2003). Salem et al. (2015) also report a similar result in their experiment. They showed that a robot that displays partially incongruent co-verbal gestures is seen to be more anthropomorphic. In our High– and Medium–Follow conditions, many participants reported that the robot was merely mimicking their responses, thus possibly making them feel that the robot's behavior is predictable and thereby decreasing its anthropomorphic element.

There was no difference in perceived agency. Since agency is tied to intellect, we speculate this is the case because the design of the experiment does not allow for the participants to evaluate the android's intelligence. In the current experiment, the participants and the android were requested to imagine an animal. In other words, there is no single correct answer to the question, thus excluding the evaluation of intelligence. We also note here that the *post-hoc* power analysis of the results seen for the perceived agency was weak (Order of Response: 1-β = 0.077, Degree of Congruence: 1-β = 0.183, Interaction: 1-β = 0.118) (Table 6). The results may not have met a statistically significant level owing to a small sample size. Thus, further data collection may be required.

To the best of our knowledge, this is the first study that has examined how sharing a representation of a certain target influences the mind attribution of a robot. Previous studies on what influences the mind attribution of a robot were more or less centered around the robot's appearance (DiSalvo et al., 2002; Powers and Kiesler, 2006; Eyssel et al., 2012; Gray and Wegner, 2012). Some results showed that robots with human-like appearance were perceived to have more mind attribution (Woods et al., 2004; Walters et al., 2008). We believe that the current study presents a new way to approach this topic, that is, introducing a method to influence the mind attribution of a robot by matching the degree of shared representation at will.

In the current experiment, we utilized hot reading to obtain the participants' representation. The hot reading refers to a technique where one gains information (birth date, names of relatives, etc.) of a certain person beforehand without the subject noticing that he/she has given up this information. This method is often employed by fortune-tellers (so-called psychics and/or palm readers) (^2^ The Sketic's Dictionary, 2015). Obtaining information for hot reading has never been easier than it is now owing to various social networking services (SNSs). Investigator can obtain participants' information through SNSs prior to the experiment and use it to their advantage. This new experimental paradigm may open new possibilities for future research, in which the participant must be convinced that the experimenter or robot shares the same reality.

While the current study aims to realize shared reality between human and robot, one can say that the matching of the representation is a form of interpersonal coordination. Interpersonal coordination refers to the synchronization of behaviors across social members (Bernieri and Rosenthal, 1991). It is well-documented in previous studies on HRI that matching behaviors (motion Shen et al., 2015, emotion Jung, 2017, and thoughts You and Robert, 2018) is known to contribute toward establishing harmonious relationships. For example, joint attention, which is the act of following the attention of others, can be observed in HRI context (Bekkering et al., 2009; Staudte and Crocker, 2011). Other studies have introduced the influence of coordinating emotions (Jung, 2017). The coordination seen in the current study was about matching of thoughts. Most of these studies on interpersonal coordination in HRI context report that coordination has a positive impact on HRI. It may be worthy to note and incorporate various coordination skills to a robot's behavior and examine how the quality of HRI experience is influenced.

### Limitations

The present study utilized a visual perception task to precisely control the order of response and degree of congruence and showed its effect on the perceived mind. However, it is not certain whether the same influence can be observed with different modalities. Further investigation is required to confirm whether the current result can be generalized to other forms of perception.

The experiment was conducted with only one type of robot. Therefore, we cannot conclude that the results of the present study can be generalized to other robots with different physical characteristics. Gray and Wegner showed that a human-like robot has higher perceived experience compared with a mechanical robot (2012). Therefore, the extremely human-like appearance of our android robot may have been an important element for the participants to perceive experience. It is worth investigating the extent of human-like appearance of the robot to replicate the present findings.

Furthermore, there was no conversation between the participants and the robot during the experiment. This demonstrates the uniqueness of the present experimental paradigm. To apply the present findings to a real-world HRI context, it is necessary to extend the experiment paradigm so that it involves a realistic interaction. For example, an interaction that involves the participant and the robot discussing their preferred paintings during an art museum tour and incorporating the matching of representations in visual perception could be arranged.

We also note here that replicating the current experiment paradigm with more linear scale may be of value. It is possible that 0 and 10~20% match rate may evoke different impression. Same can be said for 80~90 and 100%.

## Conclusions

To summarize, in the present study, we examined how the effect of matching a representation between humans and a robot influences the mind perception toward the robot. The participants and the robot observed 10 ambiguous pictures together and each imagined an animal based on the picture. The greater the congruence between the animals imagined by the human and the robot, the higher did the participants evaluate the robot's closeness in a relationship, thus confirming the results of the previous study, which showed positive correlation between having the same attitude and social bonding (Ono et al., 2001). Participants were more likely to attribute experience, which is the capacity to feel, to the robot when it stated its interpretation of the image first, compared to when it expressed its interpretation after the participant. The result suggests that the order of response evokes different perceptions of experience, and the robot expressing its idea first is more effective in in this regard. No effect of degree of congruence and the order of responses were observed in the perceived agency (the capacity to plan and do). We speculate that agency is tied to the appearance of the robot. It is possible that the extremely human-like appearance of the robot gave the impression that it possesses sophisticated intelligence to the point that it may have caused a ceiling effect on the perceived agency. Further investigation is required to confirm this hypothesis. The present experimental paradigm enables providing people with the impression of sharing the same representation at will. The paradigm opens new possibilities to conduct further research on the subject in greater depth.

## Ethics Statement

This study was carried out in accordance with the recommendations of the Graduate School of Engineering Science of Osaka University with written informed consent from all subjects. All subjects gave written informed consent in accordance with the Declaration of Helsinki. The protocol was approved by the Graduate School of Engineering Science of Osaka University.

## Author Contributions

KT, HT, YY, and HI designed the research. KT performed the research. KT conducted the analyses. KT, HT, and YY wrote the manuscript.

### Conflict of Interest Statement

The authors declare that the research was conducted in the absence of any commercial or financial relationships that could be construed as a potential conflict of interest.

## Footnotes

^1^HUIS TEN BOSCH Co., Ltd., https://youtu.be/y4ONhC6PnpI

^2^The Sketic's Dictionary (2015). http://skepdic.com/hotreading.html
